# Comment on “Expiratory time constants in mechanically ventilated patients rethinking the old concept: a narrative review”

**DOI:** 10.1186/s40635-025-00751-x

**Published:** 2025-04-18

**Authors:** Rachana Mehta, Shubham Kumar, Ranjana Sah, Edward Mawejje

**Affiliations:** 1 Department of Microbiology, RDC, Manav Rachna International Institute of Research and Studies, Faridabad, Haryana 121004 India; 2 Center for Global Health and Research, Saveetha Medical College and Hospital, Saveetha Institute of Medical and Technical Sciences, Saveetha University, Chennai, India; 3 Department of Paediatrics, Dr. D. Y. Patil Medical College Hospital and Research Centre, Dr. D. Y. Patil Vidyapeeth (Deemed-to-Be-University), Pimpri, Pune, 411018 Maharashtra India; 4 Department of Public Health Dentistry, Dr. D. Y. Patil Medical College Hospital and Research Centre, Dr. D. Y. Patil Vidyapeeth (Deemed-to-Be-University), Pimpri, Pune, 411018 Maharashtra India; 5 School of Public Health, Makerere University College of Health Sciences, Mulago Hill, Kampala, Uganda

**Dear Editor,**

We appreciate the narrative review by Depta et al. (2025) [1], published in *Intensive Care Medicine Experimental*, titled “Expiratory time constants in mechanically ventilated patients: rethinking the old concept.” The authors adeptly chronicle the evolution of the expiratory time constant (RCEXP) from a theoretical construct to a parameter with growing clinical relevance in mechanical ventilation. Their detailed examination of RCEXP’s historical roots, its limitations within the single-compartmental model, and its behavior in conditions such as acute respiratory distress syndrome (ARDS) and chronic obstructive pulmonary disease (COPD) is commendable. We also value their forward-looking perspective on integrating RCEXP into intelligent ventilation strategies, which aligns with advancements in bedside monitoring technology.

Nevertheless, we respectfully suggest that the review could be enhanced by addressing certain technical aspects more thoroughly. One area is the interplay between RCEXP and specific ventilator settings. While the authors note the influence of tidal volume (VT) and positive end-expiratory pressure (PEEP), a deeper discussion of how different ventilatory modes such as volume-controlled versus pressure-controlled ventilation alter RCEXP would be beneficial. For example, how do constant versus variable flow profiles, or the presence of spontaneous breathing efforts, modify RCEXP measurements? Such insights would bolster the review’s utility for clinicians navigating diverse ventilatory scenarios.

Additionally, the practical challenges of measuring RCEXP at the bedside merit greater attention [2]. The review outlines methods like the V’/V curve and direct expiratory flow analysis, yet it only briefly touches on confounding factors such as endotracheal tube resistance, circuit impedance, or patient positioning. These elements can significantly affect RCEXP accuracy. A discussion of how ventilator-specific algorithms or hardware variations might introduce measurement discrepancies would also be pertinent, as standardization remains a challenge in clinical practice [3]. Elaborating on these limitations could better equip readers to interpret RCEXP in real-world settings.

The clinical implications of RCEXP, while intriguing, could be substantiated further. The authors propose its use in optimizing PEEP and respiratory rate (RR), particularly in ARDS and COPD, but the evidence base for these applications appears underdeveloped [4]. For instance, how does RCEXP-guided PEEP titration compare to traditional approaches like the PEEP-FiO2 table or esophageal pressure monitoring in terms of efficacy or patient outcomes? Referencing clinical studies that validate RCEXP-based adjustments or highlighting their absence would lend greater credibility to these recommendations and clarify research priorities.

Despite these observations, Depta et al. offer a valuable synthesis of RCEXP’s role in modern ventilatory management. Their advocacy for exploring nonlinear models and regional RCEXP variations, potentially via tools like electrical impedance tomography, is a forward-thinking contribution. We encourage the authors to consider extending their analysis by comparing RCEXP to other dynamic parameters, such as driving pressure or elastance, to delineate its unique advantages.

This review is a thoughtful resource for intensivists and researchers seeking to refine mechanical ventilation strategies. By expanding on ventilator mode effects, measurement complexities, and clinical evidence, future iterations could further elevate its impact. We applaud the authors’ efforts and look forward to seeing RCEXP’s potential realized through ongoing investigation and application in critical care.

## Data Availability

Not applicable, no data was synthesized in this article.
